# Enhanced MEA Performance for an Intermediate-Temperature Fuel Cell with a KH_5_(PO_4_)_2_-Doped Polybenzimidazole Membrane

**DOI:** 10.3390/membranes12080728

**Published:** 2022-07-23

**Authors:** Yifan Li, Jing Hu, Joan Papavasiliou, Zhiyong Fu, Li Chen, Haibin Li

**Affiliations:** 1 State Key Laboratory of Ocean Engineering, School of Naval Architecture, Ocean & Civil Engineering, Shanghai Jiao Tong University, Shanghai 200240, China; lyf118811@sjtu.edu.cn (Y.L.); jinghu@sjtu.edu.cn (J.H.); xiaozhenqingnian@sjtu.edu.cn (Z.F.); li.h.chen@sjtu.edu.cn (L.C.); 2 Department of Materials Science, University of Patras, 26504 Patras, Greece; ipapavas@upatras.gr

**Keywords:** polybenzimidazole, high temperature proton exchange membrane, molten electrolyte, KH_5_(PO_4_)_2_

## Abstract

This work exhibits an effective approach to enhance the performance of membrane-electrode assembly (MEA) with KH_5_(PO_4_)_2_-doped PBI membrane, by adding phosphoric acid (PA) in the catalyst layer (CL). The ohmic resistance and single-cell performance of the MEA, treated with PA, are reduced by ~80% and improved by ~800%, respectively, compared to that of untreated MEA. Based on the MEA pretreated with PA, the influence of humidity and temperature on the resistance and the single-cell performance are investigated. Under humidified gas conditions, the ohmic resistance of MEA is reduced but the charge transfer resistance is slightly increased. Regarding the effect of temperature, the ohmic resistance of MEA becomes lower as the temperature elevates from 140 to 180 °C, but increases at 200 °C. The maximum peak power density presents at 180 °C and 20% RH with 454 mW cm^−2^. The peak power density is favored with temperature increase from 140 to 180 °C, but decreases with further increase to 200 °C. Moreover, when dry gas conditions are employed, the output performance is unstable, suggesting that humidification is necessary to inhibit degradation for a long-term stability test.

## 1. Introduction

Fuel cells are friendly to the environment and are attracting huge attention as one of the most promising technologies for efficiently converting chemical energy into electrical energy. According to the operating temperature, fuel cells can be grouped as high-temperature fuel cells (operating above 600 °C), intermediate temperature fuel cells (ITFCs) (operating between 100–600 °C), and low temperature fuel cells (operating below 100 °C) [1]. ITFCs have better tolerance to CO, simpler water management and higher electrode kinetics than low temperature fuel cells, and have less cost to prepare fuel cell devices compared to the high temperature fuel cells [2,3].

Solid acid is considered as a potential electrolyte for ITFCs. Among potential solid acid electrolytes for fuel cells operating at 200–300 °C, cesium phosphates were firstly reported by Haile [4]. The CsH_2_PO_4_ exhibits high proton conductivity in the order of 10^−2^ S cm^−1^, by converting to the superionic phase at 238 °C. The phosphate-based solid acids generally consist of alkali metals and tetrahedral oxyanion linked with hydrogen bonds, and have a formula of M_a_H_b_(PO_4_)_c_ (M = Na, K, Rb, Cs). Phosphate proton conductors based on cesium species have been extensively studied [5,6,7].

MH_5_(PO_4_)_2_ (M = Na, K, Rb, Cs), a close relative family of the MH_2_PO_4_ (M = Na, K, Rb, Cs) solid salt family, is not subject to a phase transformation into a superionic phase until heated up at the melting point, where the molten state of MH_5_(PO_4_)_2_ under humidity atmosphere leads to an increase in conductivity to 10^−2^ S cm^−1^, independent of the alkali metal species [8,9]. Unfortunately, the mechanical properties of electrolyte membrane made from pure MH_5_(PO_4_)_2_ are weak in the molten state, therefore in order to improve the mechanical properties, the electrolyte membranes need some matrix to support them. Some inorganic supports, such as SiO_2_ and SiP_2_O_7_, were reported for this purpose [10,11]. However, in order to obtain an acceptable mechanical strength and prevent gas leakage, the thickness of this composite electrolyte membrane should be up to millimeters [12,13]. Although such electrolytes composed with MH_5_(PO_4_)_2_ retain high conductivity, the area specific resistance of electrolytes was still high because the electrolyte’s thickness staying in the millimeter range. In our previous work, aiming to reduce the thickness of the electrolyte with promising mechanical strength, we reported the preparation of composite membranes based on the molten proton conductor KH_5_(PO_4_)_2_ doped within a polybenzimidazole (PBI) polymer support matrix, respectively [14]. The PBI membranes doped with molten conductor showed high proton conductivity and good mechanical properties, though this was not reflected in a good fuel cell performance. The employed membrane electrode assembly (MEA) prepared with electrolytes based on KH_5_(PO_4_)_2_ exhibited a poor peak power density of 40 mW cm^−2^ under H_2_/O_2_ [14]. Such low fuel cell performance has been a major impediment to the application of electrolyte membranes for molten proton conductors. Since the molten proton conductor hardly penetrates into CL due to the solid state at the ambient temperature, the proton conductor is difficult to migrate from electrolyte membrane to the CL during activation process. The deficiency of electrolyte content in CL results in a decrease of the active catalysis surface area [15,16], thus presenting lower MEA electrochemical performance. Depositing phosphoric acid (PA) into the CL before assembling the MEA is proposed to improve the proton conduction between the molten proton conductor doped PBI membrane and the CL, therefore expecting to improve the fuel cell performance [17,18].

In this study, we focus on using the KH_5_(PO_4_)_2_, a member of the MH_5_(PO_4_)_2_ family, as a molten proton conductor doped in the PBI membrane. Because, as compared with other membranes of the MH_5_(PO_4_)_2_ family [9,19,20], KH_5_(PO_4_)_2_ presents interesting advantages. Firstly, the proton conductivity of KH_5_(PO_4_)_2_ is greatly improved by melting at ~130 °C, and it remains stable up to 200 °C under humidified atmosphere. In this way, the operating temperature range of KH_5_(PO_4_)_2_ is active from 130 up to 200 °C. It should be mentioned that this operating temperature range matches the corresponding range of the PA-doped PBI membranes for high temperature proton exchange membrane fuel cells (HT-PEMFC), making KH_5_(PO_4_)_2_ a promising alternative proton conductor for HT-PEMFC. Secondly, the main raw material for synthesis of KH_5_(PO_4_)_2_ is K_2_CO_3_, which has lower cost than Cs_2_CO_3_ or Rb_2_CO_3_ used as raw materials of CsH_5_(PO_4_)_2_ and RbH_5_(PO_4_)_2_, respectively. The main purpose of the present work is to implement an effective approach to improve the performance of MEA assembled with KH_5_(PO_4_)_2_-doped PBI membrane. 

## 2. Experimental

### 2.1. Materials

K_2_CO_3_ (99.9%) was purchased from Shanghai Aladdin Bio-Chem Technology Co., Ltd (Shanghai, China). H_3_PO_4_ (85 wt.%) was purchased from Sinopharm Chemical Reagent Co., Ltd (Shanghai, China). The PBI membrane (the thickness is 35 μm, Fumapem®AP-30) was purchased from FUMATECH BWT GmbH (Am Grubenstollen, Germany). Gas diffusion layer (Pt loading of 1 mg cm^−2^, HT 140E) was purchased from Advent Tech (Patras, Greece).

### 2.2. Preparation of Electrolyte Membrane

Pure KH_5_(PO_4_)_2_ was synthesized by K_2_CO_3_ and H_3_PO_4_ according to the method described elsewhere [14]. To obtain KH_5_(PO_4_)_2_-doped PBI membrane, pure PBI membrane was immersed in molten KH_5_(PO_4_)_2_. Firstly, a certain amount of prepared KH_5_(PO_4_)_2_ powder was melted at a drying oven at 130 °C. Then a piece of pure PBI membrane was immersed in molten KH_5_(PO_4_)_2_ for 2 days. After the immersing process, the extra KH_5_(PO_4_)_2_ was wiped from the surface of the membrane with a non-woven paper.

The preparation process of PA-doped PBI membrane was similar to the preparation process of the KH_5_(PO_4_)_2_-doped PBI membrane. The pure PBI membrane was immersed in H_3_PO_4_ solution for 6 h, as suggested by the supplier. After immersing process, the extra PA was wiped from membrane surface with a non-woven paper, and the PA-doped PBI membrane was obtained.

### 2.3. Characterization of Membranes

The X-ray diffraction (XRD) diffractometer (Bruker, Berlin, Germany, D8 ADVANCE) with a scan rate of 6° min^–1^ was employed to evaluate the phase purity of above prepared KH_5_(PO_4_)_2_. The cross-section morphology of the membrane was investigated by scanning electron microscope (SEM) (JEOL, Tokyo, Japan, JSM-7800F), with an energy dispersive X-ray spectrometer (EDS). Thermogravimetric analysis (TGA) and differential scanning calorimetry (DSC) performed by the synchronous thermal analyzer (NETZSCH, Selb, Germany, STA449F3) were utilized to investigate the thermal properties of the KH_5_(PO_4_)_2_-doped PBI membrane. All the samples were firstly heated at 120 °C and then cooled down to 80 °C, and then, finally, heated up to 800 °C with a heating rate of 5 °C min^−1^ under air flow of 40 mL min^−1^.

The swelling ratio was determined by recording its thickness and dimension of the membrane after immersing in molten salts. Every 12 h, the membrane’s thickness and dimension were recorded after the immersed membrane was gently wiped with non-woven paper to clean the extra salt from its surface. The swelling ratio of the membrane was defined by the following equation:

(1) $$\mathrm{Swelling}\;\mathrm{ratio}\;\left(\mathrm{vol}.\%\right)=\frac{(T_{doped}\times A_{doped}-T_{dry}\times A_{dry})\times 100\%}{T_{dry}\times A_{dry}}$$

where *T_dry_* and *A_dry_* are the thickness and dimension of the pure PBI membrane, and *T_doped_* and *A_doped_* are the thickness and dimension of the immersed membrane recorded every 12 h.

The through-plane proton conductivities of KH_5_(PO_4_)_2_-doped PBI membrane were collected by an electrochemical workstation (Solartron, Leicester, England, ENERGYLAB XM,) with an amplitude of 10 mV in the frequency range from 10^−1^ Hz to 10^6^ Hz. The data of proton conductivities were recorded in the temperature range from 80 °C to 200 °C. Humidification was applied while testing proton conductivities. The method of testing conductivity was referred to the through-plane conductivity testing method described [21]. The proton conductivity (σ) was calculated by the following equation:

(2) $$\sigma =\frac{L}{AR}$$

where *A* is the electrode area, *R* is the ohmic resistance measured by workstation, and *L* is the thickness of the membranes.

### 2.4. Preparation of MEA

Three types of MEA were prepared, based on: (1) KH_5_(PO_4_)_2_-doped PBI membrane and untreated GDE, (2) KH_5_(PO_4_)_2_-doped PBI membrane and treated GDE, and (3) PA-doped PBI membrane and untreated GDE. The details of the preparation of MEAs were as follows. Firstly, for the treated GDE, the side at CL was sprayed with PA/ethanol mixture (PA/ethanol in a volume ratio of 1:5) and then dried for 30 min at 120 °C, for the untreated GDE, it was directly used without PA spraying. The amount of PA reserved in the GDE was 5 mg cm^−2^. Then, the KH_5_(PO_4_)_2_-doped PBI membrane was sandwiched between two pieces of treated GDE, and then assembled into MEA at room temperature without hot pressing. Finally, the MEA of KH_5_(PO_4_)_2_-doped PBI membrane with an active area of 5 cm^2^ was prepared, where this type of MEA was designated as PBI/K MEA. A similar preparation process was applied for the other two types of MEA. The second type of MEA was prepared with KH_5_(PO_4_)_2_-doped PBI membrane, but without PA sprayed on the surface of CL, and this type of MEA was designated as PBI/K untreated MEA. In the third type of MEA the membrane was substituted with PA-doped PBI membrane, and this type of MEA was designated as PBI/PA MEA.

### 2.5. Single-Cell Test and EIS Analysis

A fuel cell station (TOYO, Tokyo, Japan, MiniTest 3000) was employed to test performance of the single-cell. The anode was fed with dry or humidified hydrogen and the cathode with dry oxygen, the flow rate was set at 0.4 L min^−1^ at both electrode sides. Considering that the water generated at the cathode will reversely humidify the cathode, the cathode was fed with dry oxygen. On the other hand, the lack of humidity in the anode may cause electrolyte dehydration, so the anode was fed with humidified hydrogen. The latter was controlled in the range from 0% to 30% relative humidity (RH) by pumping different flow ratios of water into the hydrogen gas tube. It should be mentioned when testing the PBI/PA MEA, both hydrogen and oxygen fed in the single-cell were dry gases, because humidity has a negative effect on the performance of the PA-doped PBI membrane [22]. To guarantee that the single-cell could be fully activated, the single-cell experienced an activation process, being operated with a constant voltage of 0.6 V at 180 °C until a constant performance of the single-cell was achieved. The single-cell was evaluated by polarization measurements (current–voltage–power curves) obtained in the temperature range of 140–200 °C.

The resistance of the MEA was analyzed via galvanostatic alternating current (AC) impedance spectroscopy at atmospheric pressure in the same temperature range, employing an electrochemical workstation (Solartron, Leicester, England, ENERGYLAB XM). The frequency was set in the range of 10^−1^ to 10^6^ Hz. The direct current (DC) levels were set at 100, 200, 500, and 1000 mA. A sinusoidal perturbation of the alternating current (AC) was applied, and the amplitude of the AC was set in 5% of the DC. Electrochemical surface area (ECSA) was investigated by cyclic voltammetry (CV) between 0.1 V to 1 V at a scan rate of 0.025 mV s^−1^. The ECSA was calculated by the following formula [19]:

(3) $$\mathrm{ECSA}\;\left(m^{2}{\;g}^{-1}\right)=\frac{Q}{Q_{m}\times m_{pt}}$$

where *Q* is the adsorption charge for the electrode, *Q_m_* is the adsorption charge for a platinum surface, generally accepted to be 210 μC cm^−2^, and *m_pt_* represents 1 mg cm^−2^ of the Pt loading of GDE.

## 3. Results and Discussion

### 3.1. Characterization of KH_5_(PO_4_)_2_ and Cross-Sectional Morphology of KH_5_(PO_4_)_2_-Doped PBI Membrane

**Figure 1:**
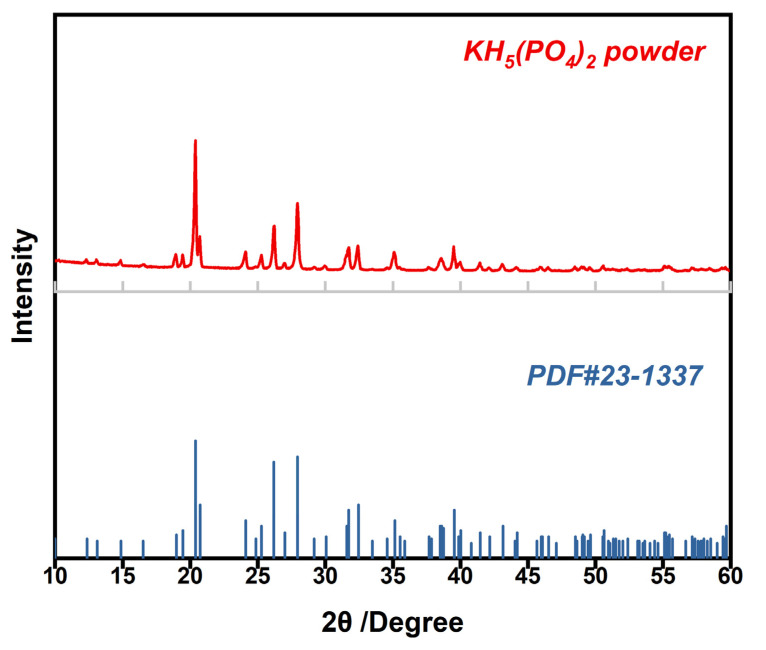
XRD pattern of KH_5_(PO_4_)_2_ powder (red color); the peak position of standard PDF card of KH_5_(PO_4_)_2_ (PDF# 23-1337, blue color).

The XRD pattern of homemade KH_5_(PO_4_)_2_ is shown in Figure 1. The peak positions of KH_5_(PO_4_)_2_ powder coincide well with those of standard PDF card for KH_5_(PO_4_)_2_, implying a high purity of the homemade KH_5_(PO_4_)_2_.

**Figure 2:**
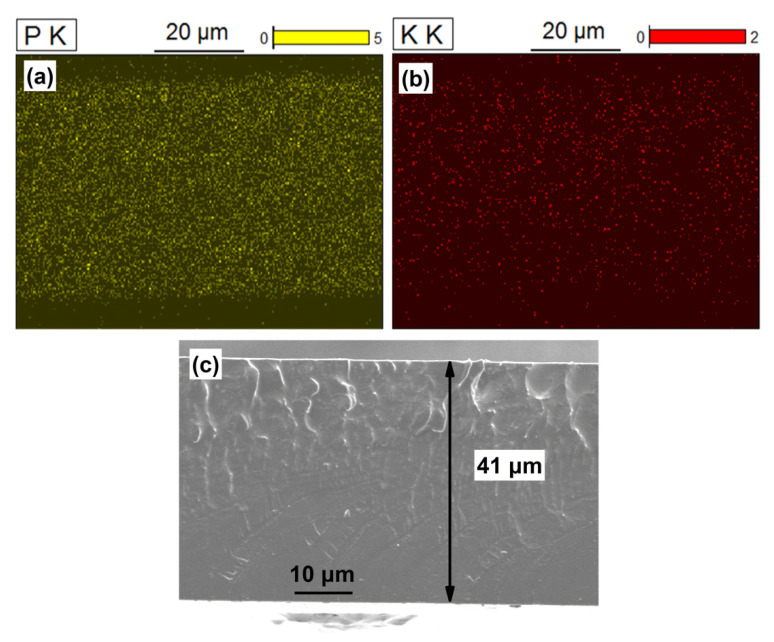
EDS mapping of (**a**) phosphorus and (**b**) potassium elements on the cross-section of KH_5_(PO_4_)_2_-doped PBI membrane. (**c**) The cross-sectional morphology of KH_5_(PO_4_)_2_-doped PBI membrane.

To confirm the PBI membrane was entirely doped with KH_5_(PO_4_)_2_, distributions of phosphorus and potassium elements, and a cross-section morphology of KH_5_(PO_4_)_2_-doped PBI membrane are shown in Figure 2. In the Figure 2a, it can be observed that the phosphorus element is uniformly distributed on the entire cross-section. Similarly, the distribution of potassium elements is also uniformly as shown in Figure 2b. Since the pure PBI membrane has no elements of phosphorus and potassium, the uniform distribution of phosphorus and potassium means that PBI membrane was fully doped with phosphate. In Figure 2c, the measured thickness of membrane is 41 μm, and the cross-section of KH_5_(PO_4_)_2_-doped PBI membrane was dense and even without pores.

### 3.2. The Swelling Ratio of KH_5_(PO_4_)_2_-Doped PBI Membrane

**Figure 3:**
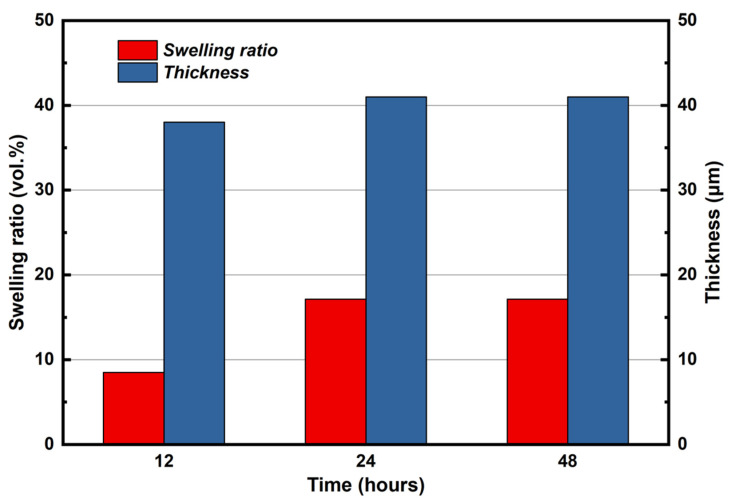
The thickness and the swelling ratio of KH_5_(PO_4_)_2_-doped PBI membrane changing with doping time.

Figure 3 shows the trending of swelling ratio and thickness of KH_5_(PO_4_)_2_-doped PBI membrane within 48 h. The swelling ratio and the thickness of membrane were increased with doping time, though not proportional. At the initial 12 h, the thickness of immersed membrane was increased from 35 to 38 μm; after immersing in molten salt for 24 h, the thickness of membrane was increased to 41 μm, and the swelling ratio reached 17 vol.%; until 48 h, both thickness and swelling ratio of immersed membrane remained unchanged. During the impregnation process, the change in the volume of the membrane was mainly caused by increasing in the thickness of membrane, and the dimension of membrane was almost unchanged, which was not shown in Figure 3.

### 3.3. Thermal and Mechanical Properties of KH_5_(PO_4_)_2_-Doped PBI Membrane

**Figure 4:**
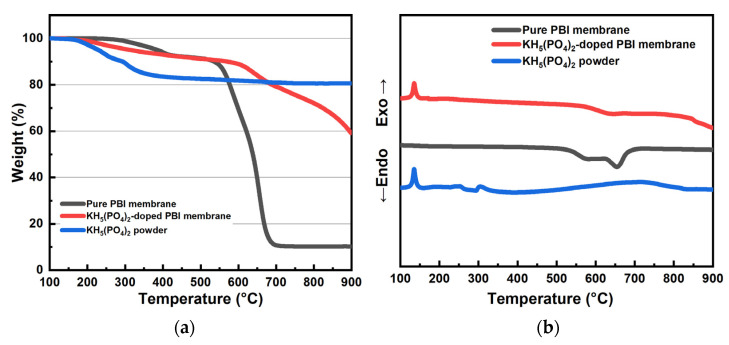
TGA curves (**a**) and DSC curves (**b**) of the PBI membrane, KH_5_(PO_4_)_2_-doped PBI membrane, and KH_5_(PO_4_)_2_ powder.

The thermal properties of the pure PBI membrane, KH_5_(PO_4_)_2_-doped PBI membrane and KH_5_(PO_4_)_2_ powder were accessed by TGA and DSC analysis, as shown in Figure 4. In Figure 4a, the TGA curve of KH_5_(PO_4_)_2_ powder indicates that the KH_5_(PO_4_)_2_ powder is stable up to ca. 200 °C, and then experiences continuous loss of weight until 400 °C, which can be attributed to dehydration of KH_5_(PO_4_)_2_, similarly with previous report [15]. For pure PBI membrane, the weight loss starts at ca. 300 °C, and presents a sharp weigh loss from 530 °C to 690 °C. This behavior is due to the decomposition taking place when PBI is heated above its glass transition temperature (T_g_ = 425–436 °C) [23]. The thermogravimetric behavior of KH_5_(PO_4_)_2_-doped PBI membrane shows two degradation periods that correspond to the dehydration of KH_5_(PO_4_)_2_ and decomposition of PBI membrane, respectively. KH_5_(PO_4_)_2_-doped PBI membrane starts its weight loss at 200 °C, similarly with KH_5_(PO_4_)_2_ powder. The weight loss continues up to 600 °C, and then the second sharp degradation occurs due to the decomposition of PBI membrane. In Figure 4b, both KH_5_(PO_4_)_2_ powder and KH_5_(PO_4_)_2_-doped PBI membrane have an endothermic peak at 130 °C, attributed to the melting of KH_5_(PO_4_)_2_-doped PBI membrane [14]. 

**Figure 5:**
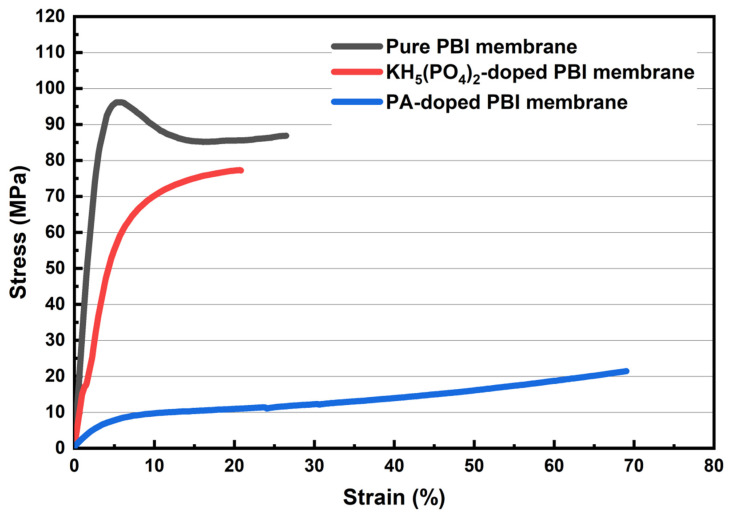
Stress-strain curves of the pure PBI membrane, KH_5_(PO_4_)_2_-doped PBI membrane, and PA-doped PBI membrane.

The stress–strain curves of KH_5_(PO_4_)_2_-doped PBI membrane, PA-doped PBI membrane, and pure PBI membrane are shown in Figure 5. It can be observed that the pure PBI membrane obtains the highest tensile strength at 96 MPa compared with the other two electrolyte membranes. The PA-doped PBI membrane shows the lowest tensile strength at 22 MPa. Such low mechanical properties can be due to the fact that the PBI membrane doping with PA is swollen to ca. 200 vol.% [24] and the intermolecular force between polymer chains of PBI membrane is weakened by PA [25]. The doping process also reduced the mechanical strength of KH_5_(PO_4_)_2_-doped PBI membrane, but the maximum tensile strength was measured to be 77 MPa, which is much higher than PA-doped PBI membrane. This phenomenon could be related with the low swelling ratio of KH_5_(PO_4_)_2_-doped PBI membrane as shown in Figure 3, which is much lower than that of PA-doped PBI membrane which can reach 150–250% [26].

### 3.4. Proton Conductivity of KH_5_(PO_4_)_2_-Doped PBI Membrane

**Figure 6:**
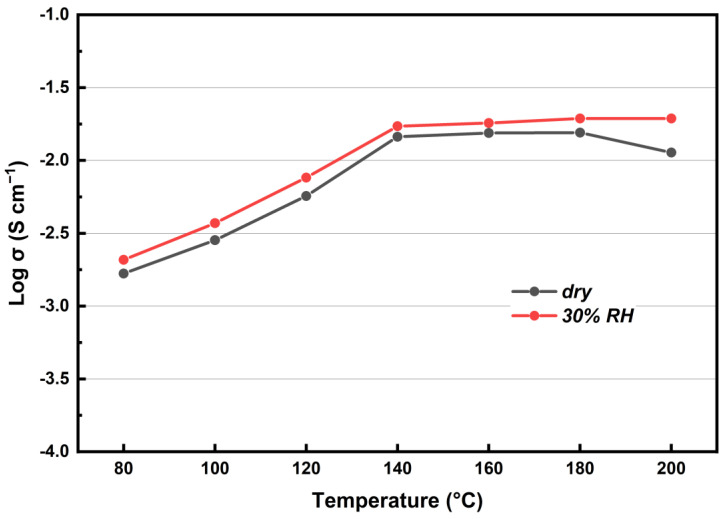
Proton conductivities of the KH_5_(PO_4_)_2_-doped PBI membrane in temperature range from 80 °C to 200 °C.

The proton conductivity of KH_5_(PO_4_)_2_-doped PBI membrane in the temperature range from 80 °C to 200 °C under dry and humidified gas conditions was evaluated. As shown in Figure 6, the proton conductivity increases with temperature in any gas conditions. From 120 °C to 140 °C, a sharp increment occurs, which is due to the melting point of KH_5_(PO_4_)_2_ at ~130 °C [14]. Under dry gas conditions, the maximum conductivity appears when the temperature rises up to 180 °C, but as temperature continues to increase, the conductivity decays, due to dehydration of phosphate salt [10,27]. In contrast, there is no decay of conductivity under humidifying conditions, which inhibits the dehydration of KH_5_(PO_4_)_2_. Moreover, in the temperature range of 80–200 °C, the conductivity under humidified gas conditions is higher than that under dry gas conditions. This can be attributed to good hygroscopicity of KH_5_(PO_4_)_2_, so that the absorption of water not only inhibits the dehydration of KH_5_(PO_4_)_2_ but also establishes extra proton conduction channels for KH_5_(PO_4_)_2_-doped PBI membrane. The higher water content absorbed in the electrolyte, leads to higher ionic mobility and renders it more conductive [28].

### 3.5. Performance of MEA before and after Treating GDEs with PA

**Figure 7:**
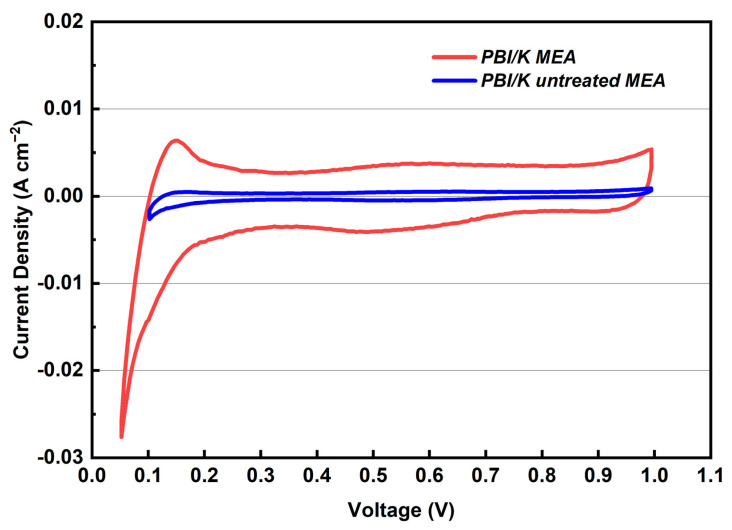
Cyclic voltammograms of treated and untreated PBI/K MEAs at 160 °C feeding with dry H_2_/N_2_ (equal H_2_ and N_2_ flow rates at 0.4 L min^−1^).

The application of this membrane was hindered due to the low performance of the MEA [14]. In this study, an effective approach by adding the PA on the surface of CL to improve the performance of KH_5_(PO_4_)_2_-doped PBI membrane is presented. To verify the effect of added PA on the performance of the MEA, three types of MEAs (PBI/K MEA, PBI/K untreated MEA, and PBI/PA MEA) were tested. In Figure 7, the corresponding CVs are presented in order to investigate this effect on the ECSA. ECSA means the contact area between Pt catalyst and proton conductor in the CL, whereas poor ECSA values could reduce the performance of MEA. ECSA values were calculated from the hydrogen desorption peaks which generally appear at a low voltage region of 0.1–0.3V. The measured ECSA values of treated and untreated PBI/K MEAs are 27.7 m^2^ g^−1^ and 0.8 m^2^ g^−1^, respectively. Such a low ECSA value of the untreated MEA indicates that the proton conductor, i.e., KH_5_(PO_4_)_2__,_ in KH_5_(PO_4_)_2_-doped PBI membrane, can hardly migrate into CL during the activation process, resulting in the lacking of proton conductor at CL, and, thereby, in insufficient triple-phase boundary in the electrode [29]. Proton conductor content in the CL is an important factor to build the triple-phase boundary between CL and membrane, which can affect the whole oxidation/reduction reactions. Before treating with PA, the absence of proton conductor content limits the proton mobility from membrane to the electrode. Since the surface of KH_5_(PO_4_)_2_-doped PBI membrane remains dry without extra phosphate salt and the proton conductor KH_5_(PO_4_)_2_ can hardly migrate into the CL, ECSA is reduced by the solid–solid contact between membrane and CL, which results in an increase in ohmic resistance. For MEA treated with PA, the solid–liquid contact between membrane and CL was established by liquid state PA, which results in enlarged triple-phase reaction zone and higher ECSA, referring to the same catalyst loading of the electrode.

**Figure 8:**
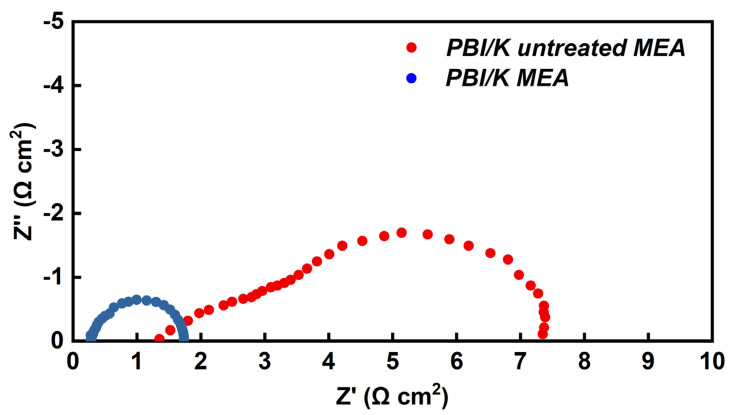
Nyquist plots of treated and untreated PBI/K MEAs at 20 mA cm^−2^ of output DC density at 160 °C, feeding with dry H_2_/O_2_ (equal H_2_ and O_2_ flow rates at 0.4 L min^−1^).

The effects of added PA on the resistance of MEAs for KH_5_(PO_4_)_2_-doped PBI membrane, was also investigated via AC impedance spectroscopy at 160 °C with 20 mA cm^−2^ of output DC density. Figure 8 depicts the comparison of the Nyquist plots between treated and untreated PBI/K MEAs. In the former case, the first intercept of the arc on the real axis represents the total ohmic resistance of the single-cell, which contains the body resistance of the various assembled parts. As compared with the membrane resistance and the contact resistance, the electrical resistance of the assembled parts can be ignored. Therefore, the first intercept on the real axis represents the ohmic resistance corresponding to the sum of the contact resistance between the membrane and the CL, and ionic resistance of the electrolyte membrane (membrane resistance, R_m_). The obtained values for the ohmic resistance of treated and untreated MEAs are 0.25 and 1.5 Ω cm^2^, respectively. Taking into account the fact that a similar process was applied for the assembly electrodes with membranes, thus there should be no difference in the ionic resistance of membranes at the same temperature, so the main difference originates from the contact resistance between membrane and CL. By treating PBI/K MEA with PA, the ohmic resistance is greatly reduced by ~80%. The fact is that the viscosity of KH_5_(PO_4_)_2_ is much higher than that of liquid phosphoric acid, and thereby the contact interface between CL and membrane with mobile liquid state of PA had lower contact resistance, in contrast to the characterized state of the contact interface of membrane and CL filled with KH_5_(PO_4_)_2_, which is in molten state. Moreover, in the high frequency region, the curve of untreated MEA presents a 45° slope, which can be attributed to the restriction of proton conduction in the CL [30]. In contrast, the high frequency region of treated MEA presents a semicircle arc, which suggests a well proton conduction in the CL due to added PA.

**Figure 9:**
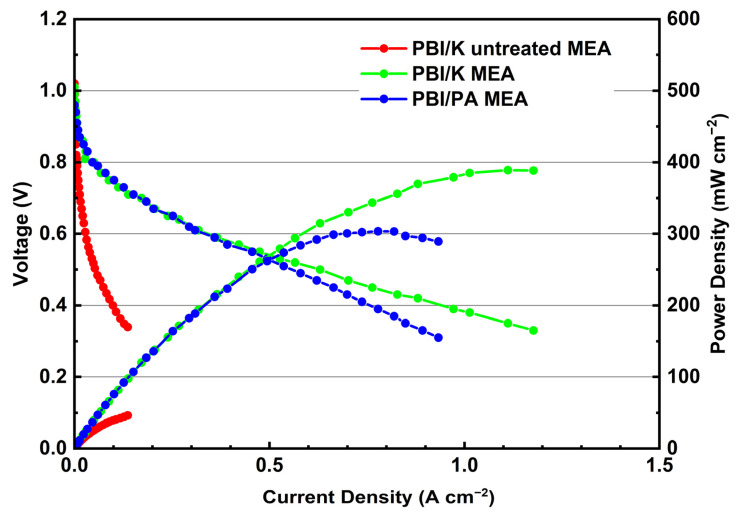
Polarization curves of treated and untreated PBI/K MEAs and PBI/PA MEA at 160 °C feeding with dry H_2_/O_2_ (equal H_2_ and O_2_ flow rates at 0.4 L min^−1^).

The electrochemical performance of treated and untreated PBI/K MEAs, at 160 °C, is presented in Figure 9. To evaluate the applicability of PBI/K MEA, the commercially available PBI/PA MEA, already applied in HT-PEMFC, is also added in Figure 9 for comparison reasons. Due to negative influence of humidified atmosphere in the performance of PBI/PA MEA [31], dry feed streams with H_2_ and O_2_ were employed in all the single-cell test. The open circuit voltage (OCV) for treated and untreated PBI/K MEAs, and PBI/PA MEA, is depicted in Figure 9, and is 1.02 V, 1.01 V, and 0.96 V, respectively. The OCV of PBI/PA MEA is lower than those of MEAs based on KH_5_(PO_4_)_2_-doped PBI membrane regardless of treating with PA. The higher OCV of MEAs based on KH_5_(PO_4_)_2_-doped PBI membrane can be attributed to the better mechanical properties and lower swelling ratio as discussed in the previous part. Taking a closer look at the polarization curves, following the current density increase, one can distinguish three types of losses that affect the gradient of the voltage drop: activation loss at high voltage region (from 0.7 V to OCV), ohmic loss at medium voltage region (from 0.4 V to 0.7 V), and mass transmission loss at low voltage region (below 0.4 V). By comparing the I-V curve of PBI/K MEA with that of untreated MEA, the latter one presents a sharper decrease in the gradient of the voltage drop in high and medium voltage regions. This behavior originates from the poor interface contact between CL and membrane of untreated MEA, which can increase the ohmic loss and enhance the lack of proton-conducting in CL that increase the activation loss, as reflected in Figure 8. Taking into account the huge difference in activation and ohmic losses, the performance of treated PBI/K MEA can be improved by ~800%. By comparing I-V curves of treated PBI/K MEA with commercial PBI/PA MEA, it is obvious that the received polarization curves are almost identical at low and medium current densities, which indicates that the ohmic resistance of treated PBI/K MEA is improved by adding PA. The discrepancy of I-V curves occurs in high current densities, probably to migration of the proton conductor PA of the PA-doped membrane from membrane to the CL, though excessive PA added in the CL could block the pores of microporous layer, and thereby increase mass transmission loss [18].

### 3.6. Performance and EIS Measurements of MEA Assembled with KH_5_(PO_4_)_2_-Doped PBI Membrane

**Figure 10:**
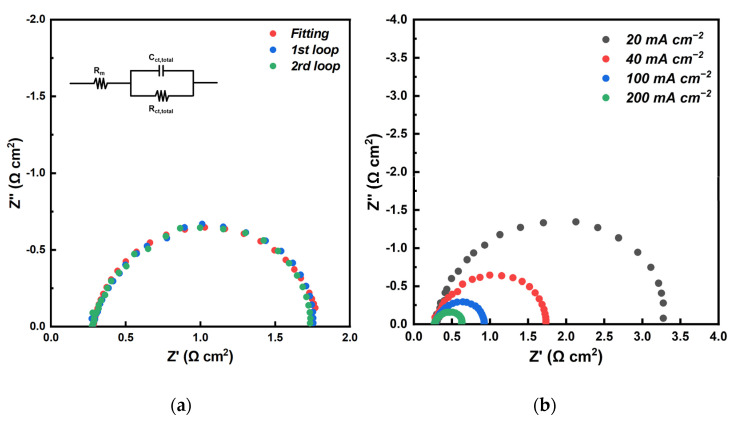
(**a**) Nyquist plots for two loops and for the fitting plot of the equivalent circuit under 0% RH gas conditions, with current density of 20 mA cm^−2^. (**b**) Nyquist plots for different current densities under 0% RH gas conditions. (at 160 °C, equal H_2_ and O_2_ flow rates at 0.4 L min^−1^).

Figure 10 illustrates the results of EIS measurements of treated PBI/K MEA at 160 °C under both dry H_2_ and O_2_ feed streams. The equivalent circuit is also included in Figure 10a. The first and second loops of EIS measurements of MEA are almost matching, indicating good repeatability. Since anode and cathode of the single-cell were fed with excess pure hydrogen and excess pure oxygen, the polarization effect of the anode was minimized and the electrode process was mainly driven by an oxygen reduction reaction (ORR) process, so that the shape of Nyquist plots in Figure 10a presents a semicircular structure, which can be simulated by Randles model [32]. Moreover, mass transfer effect of MEAs is not discussed in this study, because the area of MEAs is 5 cm^2^, and the voltage of the single-cell cannot drop below 0.6 V due to the upper limit of 2 A of the used electrochemical workstation during EIS measurements, so that the mass transfer effect normally occurring at low voltage range is considered as negligible. As shown in the equivalent circuit inserted in Figure 10a, ohmic resistance, total charge transfer resistance, and CL capacitance are represented as R_ohmic_, R_ct,total_, and C_ct,total_, respectively. It should be mentioned that the ohmic resistance is the sum of membrane resistance and contact resistance, but as the measurement of proton conductivities in Figure 6, and the area resistance of the KH_5_(PO_4_)_2_-doped PBI membrane is close to the ohmic resistance of the MEAs, thus meaning that the effect of contact resistance could be negligible, therefore the first intercept on the real axis can be regarded as R_m_. In Figure 10a, the fitting plot coincides with the results obtained from EIS, so this equivalent circuit is appropriate for MEAs assembled with KH_5_(PO_4_)_2_-doped PBI membrane. In Figure 10b, Nyquist plots under different current densities present a semicircular structure, and mass transfer effects and the anode polarization effects are not observed.

**Figure 11:**
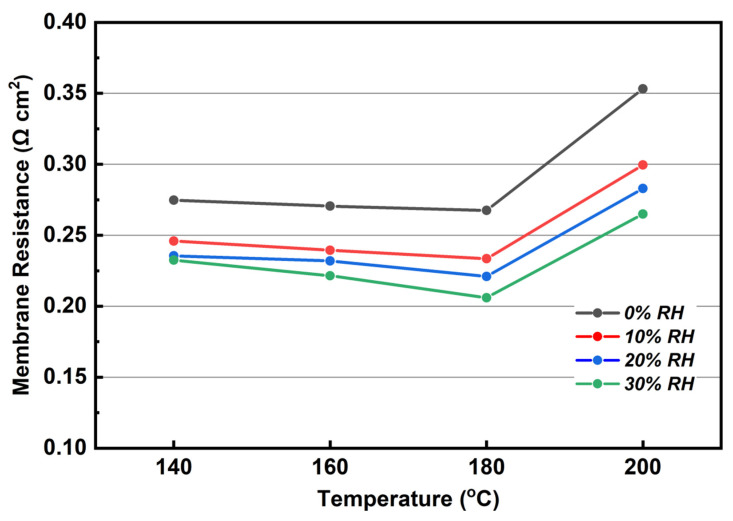
R_m_ for different RH at the anode with 20 mA cm^−2^ of output constant DC density under 0% RH gas conditions (140 to 200 °C, equal H_2_ and O_2_ flow rates at 0.4 L min^−1^).

Figure 11 shows the change of R_m_ with different RH in the temperature range of 140–200 °C. Under RH changing from 0% to 30%, the R_m_ is gradually decreased with temperature increase. This observation can be ascribed to the fact that the conductivity of the molten proton conductor increases with increasing temperature, and this trend of conductivity is represented in Figure 6, but when the temperature rises up to 200 °C, the R_m_ increases sharply independent of RH. It is speculated that due to the dehydration of phosphate, the decrease in the conductivity of the proton conductor leads to the increase in R_m_. The decrease in the conductivity of proton conductor caused by the increase in temperature is consistent with what was reported by Matsui et al. [33]. From the perspective of R_m_ changing with RH, it can be commented that under all the studied temperatures, the R_m_ decreases as the RH increases, a behavior attributed to the hygroscopicity of KH_5_(PO_4_)_2_. The absorbed water in membrane could provide extra channels for proton transfer which lead to the decrease of R_m_. By comparing the difference of R_m_ between 0 and 10% RH, it can be found that the difference at 140, 160, 180, and 200 °C is 28.8, 31, 34, and 43.7 mΩ cm^2^, respectively. For the difference of R_m_ between 10 and 30% RH, the corresponding values at above temperatures are 13.5, 18, 27.5, and 34.5 mΩ cm^2^, respectively. Obviously, the difference in R_m_ between 0 and 10% RH gradually increases as the temperature increases, which means that the humidity dependence of PBI/K MEA for keeping low R_m_ increases with elevating temperature. The difference of R_m_ between 10 and 30% RH becomes smaller than that in the lower RH range. In other words, based on the 10% RH conditions, a further increase in humidity has a smaller drop of R_m_. Meanwhile, a further increase in humidity results in lower performance of the treated PBI/K MEA, which will be discussed later.

**Figure 12:**
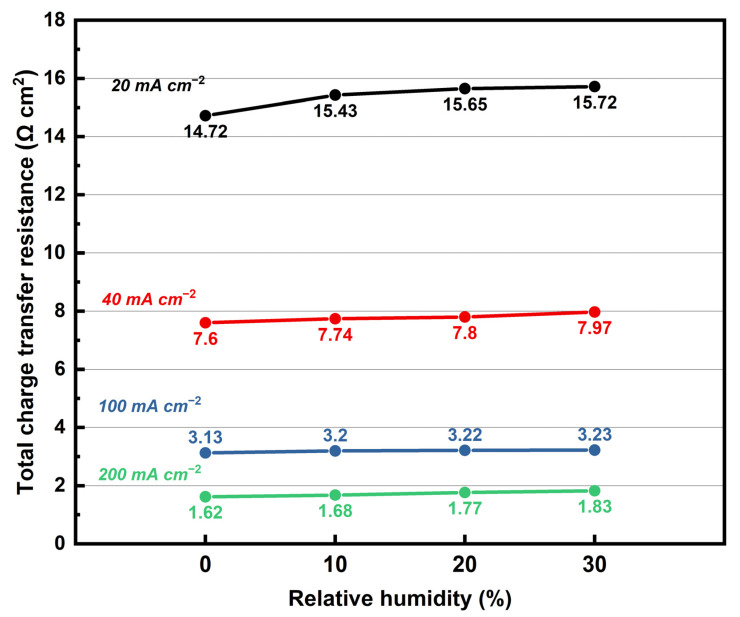
Total charge transfer resistance changing in levels of RH from 0 to 30% for different output constant DC densities (180 °C, equal H_2_ and O_2_ flow rates at 0.4 L min^−1^).

Figure 12 presents the change of R_ct,total_ with different levels of relative humidity at 180 °C, for different output constant DC densities. It can be observed that as the output DC density increases, the total charge transfer resistance decreases. According to the Butler–Volmer equation, the rate of oxygen reaction at the cathode will increase as the output DC density increases, which will reduce R_ct,total_ of the fuel cell [34]. Under all DC levels, the increase in RH results in a slight increase in R_ct,total_. This may be due to the increase in humidity of gas causing an increase in the water content of the membrane, so the absorbed water molecules at the anode convert proton into hydronium, thus contributing to an increase in the proton transmission by vehicular mechanism. Therefore, the water molecule accumulates at the cathode by carrying proton from anode to cathode, which causes an increase in the charge transfer resistance of the cathode. Similar behavior has been reported in the previous work [35].

**Figure 13:**
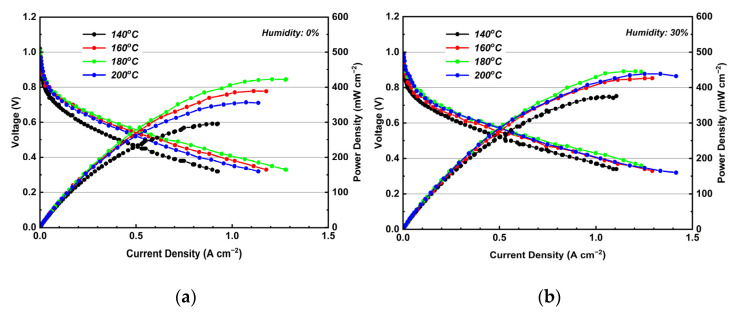
(**a**) I-V-P curves of treated PBI/K MEA at 140–200 °C under (**a**) dry and (**b**) 30% RH gas conditions. (Equal H_2_ and O_2_ flow rates at 0.4 L min^−1^).

The I-V-P curves of treated PBI/K MEA at 140–200 °C under 0 and 30% RH gas conditions are shown in Figure 13a,b. In Figure 13a, OCVs at all temperature ranges can be sustained over 1.0 V, with the highest value reaching 1.02 V. Such a good OCV can be attributed to the dense structure of KH_5_(PO_4_)_2_-doped PBI membrane, which has low permeability of hydrogen. In Figure 13b, the OCV under 30% RH gas conditions at 140, 160, 180, and 200 °C is 1, 0.99, 0.98, and 0.99 V, respectively. The treated PBI/K MEA experiences under humidified gas conditions a slight decrease in OCV, because the hydrogen partial pressure becomes lower due to the dilution of hydrogen by co-fed water vapors. This behavior can be further explained via the Nernst equation:

(4) $$E=E^{0}+\frac{RT}{2F}\ln\left(\frac{P_{H_{2}}P_{O_{2}}^{1/2}}{P_{H_{2}O}}\right)$$

As shown in Figure 13a, it can be observed that from 140 to 180 °C, the peak power density increases with increasing temperature, which is due to the fact that the increase in temperature results in an increase in the conductivity of the molten proton conductor and an improvement in the cathode dynamics. When the temperature reached 200 °C, the degradation in peak power density took place. This degradation can be attributed to dehydration of the molten proton conductor, which leads to an increase in R_m_. The trend of R_m_ changing with temperature, as illustrated in Figure 12, can reflect this phenomenon. In Figure 13b, the peak power density under 30% RH gas conditions at 140, 160, 180, and 200 °C is 374, 426, 445, and 432 mW cm^−2^, respectively. Compared with the corresponding values under dry gas conditions, the former values are higher, and this is due to the conductivity of the phosphate is improved under humidified gas conditions. Moreover, a performance comparison of the single cells with the performance of KH_5_(PO_4_)_2_-doped PBI membrane and other PBI-based composite membrane was shown in Table 1.

**Figure 14:**
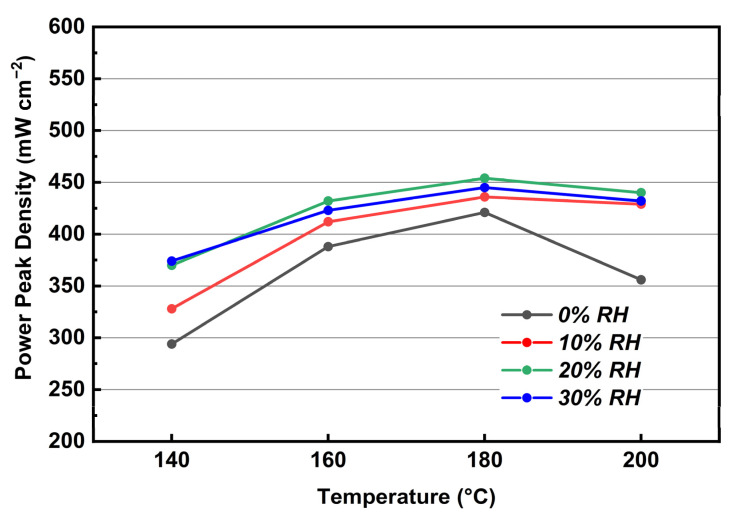
Peak power density at 140–200 °C under various levels of relative humidity. (Equal H_2_ and O_2_ flow rates at 0.4 L min^−1^).

In Figure 14, it can be observed that the peak power densities increase as the humidity increases from 0 to 20% RH gas conditions in the whole temperature region, but decreases as the humidity continues to increase to 30% RH. Such a behavior comes from the hydrogen concentration decrease as the pressure of water vapor increases, which leads to higher mass transfer losses in the anode. The maximum peak power density of MEA is presented under 20% RH gas conditions at 180 °C, achieving a value of 454 mW cm^−2^. It should be mentioned the difference of peak power densities between 0 and 10% RH gas conditions at 140, 160, 180, and 200 °C is 34, 24, 15, and 73 mW cm^−2^, respectively. Obviously, the largest difference of peak power densities occurs at 200 °C. It is speculated that the dehydration process of phosphate is promoted by the elevated temperatures. At 200 °C, the humidified gas conditions not only improve the conductivity of KH_5_(PO_4_)_2_, but also inhibit its dehydration, so that the increment in the MEA performance is more susceptible to changes in humid gas conditions from 0 to 10% RH.

### 3.7. Durability of PBI/K MEA

**Figure 15:**
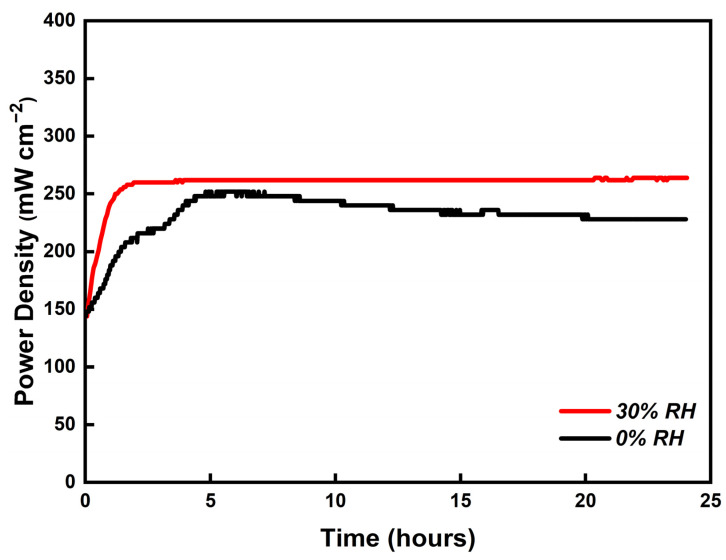
Stability tests of the single-cell with PBI/K MEA operating at 180 °C under 0 (black line) and 30% (red line) RH for 24 h (equal H_2_ and O_2_ flow rates at 0.4 L min^−1^).

To evaluate the stability of the PBI/K MEA, a 24 h durability test was carried out at 180 °C with 0 and 30% RH gas conditions, respectively. Figure 15 shows the stability trend of output power density at a constant output voltage of 0.6 V. The red line represents the trend of the output power density under 30% RH gas conditions. A gradual increase from 150 to 264 mW cm^−2^ at initial 2 h can be observed, and then a plateau at 264 mW cm^−2^ until 24 h. In the first 2 h, the output power density increases with time due to the activation of the fuel cell. The black line represents the trend of the output power density under dry gas conditions, where the obtained behavior can be divided into two periods. In the first period, the output power density increases to 250 mW cm^−2^, due to the activation of the fuel cell, followed by a gradual decrease down to 225 mW cm^−2^. Such a behavior originates from the absence of humidity atmosphere leading to the dehydration of PBI/K MEA. These results demonstrate that the humidified gas conditions can ensure a stable output power.

## 4. Conclusions

Enhancement of the electrochemical behavior of an MEA assembled with KH_5_(PO_4_)_2_-doped PBI membrane, has been achieved. Compared with the MEA untreated with PA, the applied procedure confirmed that the R_m_ of the PBI/K MEA was greatly reduced by ~80% and the ECSA was improved by about thirty times. As a consequence, the electrochemical performance of the PBI/K MEA was promoted by ~800%. All MEAs assembled with KH_5_(PO_4_)_2_-doped PBI membrane kept OCV sustained over 1 V, which can be attributed to the excellent mechanical and thermal properties of KH_5_(PO_4_)_2_-doped PBI membrane.

The effects of humidity and temperature on the ohmic resistance and charge transfer resistance were studied via EIS analysis of PBI/K MEA. The former resistance was reduced but the latter resistance was slightly increased with higher RH gas conditions. Regarding the effect of the temperature, the ohmic resistance was reduced as the temperature increased from 140 to 180 °C, but further increase at 200 °C resulted to higher resistance due to the dehydration of KH_5_(PO_4_)_2_-doped PBI membrane. 

The maximum peak power density of the PBI/K MEA appears at 180 °C, under 20% RH, with a measured value of 454 mW cm^−2^. The peak power density follows the increase in temperature from 140 to 180 °C, but further increase to 200 °C, resulted in a decrease. In addition, the stability test of PBI/K MEA indicated that the output performance is unstable under dry gas conditions. The humid gas conditions were necessary to inhibit degradation of output performance in long-term operation.

In conclusion, this work demonstrated the functionality of a simple and easily reproducible method for the preparation of MEAs with KH_5_(PO_4_)_2_-doped PBI membrane, providing a promising candidate for ITFCs at 140–200 °C.

## Figures and Tables

**Table 1:** Comparison of the performance of some PBI-based composite membrane.

Electrolyte	Proton Conductor	Pt Loading for Anode and Cathode	Operating Conditions	Peak Power Density	Open Circuit Voltage	Reference
KH_5_(PO_4_)_2_-doped PBI membrane	KH_5_(PO_4_)_2_	1 mg cm^−2^	Feeding with H_2_/O_2_ at 180 °C	450 mW cm^−2^	1.0 V	This work
PBI/GO composite membrane	PA	1 mg cm^−2^	Feeding with H_2_/air at 165 °C	380 mW cm^−2^	0.95 V	[36]
PBI/dicationic ionic liquids composite membrane	PA	0.5 mg cm^−2^	Feeding with H_2_/O_2_ at 180 °C	700 mW cm^−2^	0.85 V	[37]
PBI/TiO_2_ composite membrane	PA	0.5 mg cm^−2^	Feeding with H_2_/O_2_ at 175 °C	425 mW cm^−2^	0.85 V	[38]

## Data Availability

Not applicable.
